# Climate change: A pointer to increased small-scale fisher drowning deaths

**DOI:** 10.1371/journal.pone.0302397

**Published:** 2024-05-22

**Authors:** Ranaivo A. Rasolofoson, Horace Owiti Onyango, Fonda Jane Awuor, Christopher Mulanda Aura, Kathryn J. Fiorella

**Affiliations:** 1 Duke University Marine Laboratory, Nicholas School of the Environment, Duke University, Beaufort, North Carolina, United States of America; 2 Department of Public and Ecosystem Health, Cornell University, Ithaca, New York, United States of America; 3 School of the Environment, University of Toronto, Toronto, Ontario, Canada; 4 Department of Natural Resources and the Environment, Cornell University, Ithaca, New York, United States of America; 5 Kenya Marine and Fisheries Research Institute, Kisumu, Kenya; Surf Life Saving Australia, AUSTRALIA

## Abstract

Drowning is an overlooked public health concern and drowning risk is dependent on environmental risk factors. The preponderance of drowning deaths occurs in low- and middle-income countries. Small-scale fishers face high occupational risk of drowning. Climate change increases the frequency and intensity of storms, thereby exacerbating fishers’ risks and creating a need to examine the contribution of storms to fisher drowning deaths for the development of mitigation strategies. We examined this relationship between weather and fisher drowning deaths in Lake Victoria, which is Africa’s largest lake, a site of high fishing pressure, and where climate change is predicted to increase thunderstorms. We conducted a verbal autopsy with people knowledgeable about recent fatal fisher drowning incidents to collect information about the deceased fishers and circumstances surrounding the incidents across 43 landing sites in the Kenyan shore of Lake Victoria. Semi-structured interviews with stakeholders also elucidated community perspectives on drowning risks. Fatal drownings were often attributed to bad weather (41.8%). Other risk factors, such as non-use of life jacket and navigation equipment, co-occurred with bad weather at high rates (69.5% and 67.8%, respectively) to jointly contribute to fatal drowning incidents. Such co-occurrence of risk factors indicates that actions across multiple risk factors can help mitigate the issue. Stakeholder analysis revealed a range of opportunities for improved communication of risks and action to mitigate risks across boat operators and manufacturers, as well as multiple levels of management. Across global small-scale fisheries, limited use of safety equipment and intensive fishing pressure may coincide with increases in extreme weather events, necessitating action to address current and mitigate future drowning risks to small-scale fishers.

## Introduction

Drowning is a widely overlooked public health threat and a growing environmental issue, annually killing an estimated 236,000 people around the globe [1]. It is the third leading cause of unintentional injury mortality, accounting for 7% of the world’s injury related deaths [1]. Around 90% of unintentional drowning deaths occur in low- and middle-income countries, with the highest estimated death rates occurring in Africa [1, 2]. In some African communities located near water, drowning death rates exceed those of well-known public health threats, such as malaria, HIV, or tuberculosis [3]. However, unlike these well-known threats, drowning has received little attention in academia, practice, and policy [2, 4], particularly in low-income countries [5]. While drowning mortality is typically highest in children [6, 7],occupational groups such as fishers are often at extremely high risk as well.

Climate change is projected to increase the frequency and intensity of extreme weather events [8], with East Africa particularly vulnerable to thunderstorms [9]. While little is known about how climate-related increases in storms are already altering the incidence of drowning deaths among fishers, stormy weather conditions are an established drowning risk factors for fishers, with significant reported cases from East Africa [2, 3, 10, 11]. For example, a recent study in Lake Victoria, Africa’s largest lake, indicates an estimated 1,500 drowning deaths occur annually, of which two-thirds (1,000) are estimated to be weather related [12]. These weather shifts are therefore predicted to compound the risk of drowning for small-scale fishers in low-income countries [13, 14], who already face a range of occupational drowning risk factors: lack or inadequacy of safe boats, little life-saving equipment (e.g., life preservers), and weak safety regulation enforcement [2, 3, 10, 11]. Information on the role of stormy weather conditions, alongside other risk factors, is thus needed to shed light on the potential contribution of climate change to mortality in small-scale fishing communities and proactively design climate-related strategies (e.g., improved storm warning systems) to mitigate risks.

Lake Victoria has high fishing pressure from approximately 200,000 fishers operating on the lake [15] and is a hotspot for severe thunderstorms [16, 17] and therefore, claimed to be among the most dangerous stretches of water in the world [13, 18]. Thunderstorms over Lake Victoria are predicted to become more intense, including the intensity of precipitation and wind gusts, and up to 10 times more frequent by the end of the century [13, 14]. In 2018, on the Tanzanian side of Lake Victoria, fisher drowning death rates were estimated at a staggering 1.4%, a rate that exceeds that of even other high-risk groups (e.g., children, boat passengers) [3].

Compounding climatic effects is a change of fishing pattern over the latest few decades. As Nile perch (*Lates niloticus*), a key commercial fish species, has declined [19], fishers have increasingly turned to a sardine-like species omena (*Rastrineobola argentea*) [20]. Omena are fished far offshore, and at night when lights are used to illuminate their food sources and thereby attract fish [21]. However, thunderstorms are most likely to occur at night [13], and visibility is limited, increasing the risk of collision and poor navigation, all of which make fishers more vulnerable to drowning. Further, as fishers are often key providers within their households, pressure to fish, even in poor weather conditions to bring in needed income, can be high and drowning deaths can also have far-reaching, negative socio-economic consequences for households [3]. The interactions between climate change, vulnerability, pressure to fish, and fish declines create a negative feedback loop or socio-ecological trap that exacerbates fishers’ drowning death risks.

In the Lake Victoria region, fishing provides substantial income and health benefits locally in context where vulnerability to food insecurity is widespread [22, 23]. Fishing is interrelated with the health and well-being of local fishers who alter fishing practices in response to illness [22]. Fishers and traders on the shores of Lake Victoria have faced a longstanding risk of HIV [24, 25] with transactional sexual exchanges exacerbating risks [20, 26, 27]. Today, HIV prevalence within fishing communities along the shores of Lake Victoria remains some of the highest in the world [28]. This omnipresent health risk has been described as intertwined within a ‘sub-culture’ of risk where fishers risk their lives on the water as well [29] and may play a role in motivating fishers to go out even in risky weather conditions.

We investigated reports of drowning deaths among fishers in the Lake Victoria fishery to better understand contributing risk factors, and particularly the role of extreme weather on occupational drowning deaths in the small-scale fishery around the Kenyan shores of the lake. We also examined stakeholder perceptions of drowning risks and opportunities to mitigate them to inform future programmatic and policy efforts. The small-scale fisher dependence on fisheries and escalating climate impacts within Lake Victoria may be emblematic of small-scale fishing communities in low-income countries facing increasing storms alongside consistent pressure to fish.

## Materials and methods

### Study area

**Fig 1:**
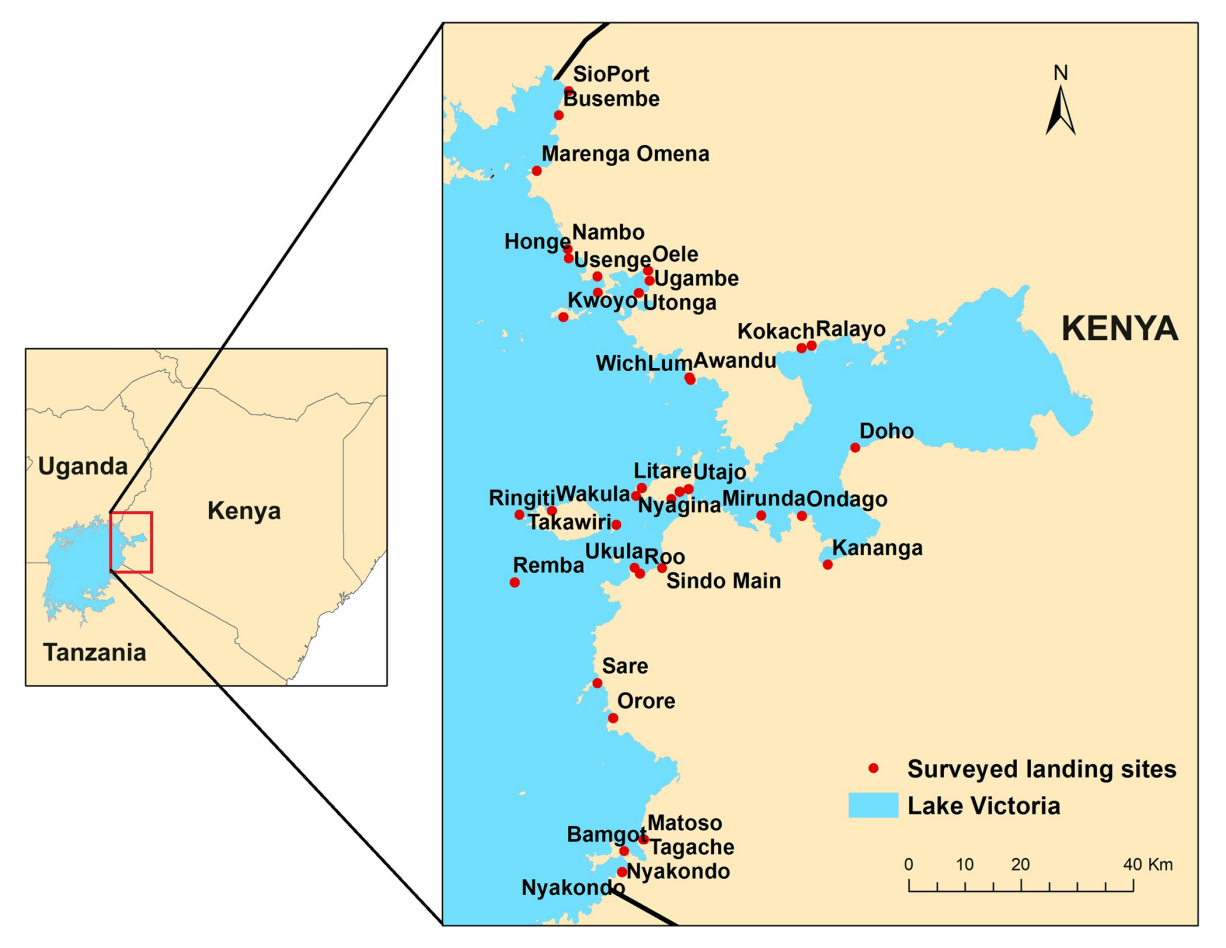
Study site: Kenyan shore of Lake Victoria (made with Natural Earth and Hamilton [36]).

Lake Victoria is shared by three countries in Eastern Africa: Kenya, Tanzania, Uganda (Fig 1). Within all three nations, small-scale fishing is widespread, and the lake fishery supports the food security and livelihoods of about 40 million people living in its basin [30, 31]. The introduction of non-native Nile perch into Lake Victoria in the 1960s precipitated rapid growth that brought migration, infrastructure development, and fish international export markets to lakeshore communities beginning in earnest by the 1980s [32–34]. The more recent decline of the Nile perch fishery has given way to expanding fishing effort to harvest omena [35].

We focus our study in the Kenyan side of Lake Victoria (Fig 1) where the lake provides for at least 76% of national fish catches and 0.4% of the country’s GDP according to official fisheries statistics [37], with estimates suggesting this may be a substantial undervaluing of the fishery [38]. The fishery in Lake Victoria provides direct employment to at least 48,000 fishers within Kenya’s sector of the lake [30].

### Sampling and data collection

This study utilized secondary data from the 2020 Lake Victoria Frame Survey to design the sampling strategy [30]. The Frame Survey is a biennial census-based approach in which fishing effort data (fishers/crew, vessels, and gears) is collected on all operators in designated fish landing sites. The survey also collects supplementary fishing information such as fishing trip dynamics, gear operation and socio-demographic information on fishers and landing facilities. The Frame Survey 2020 included a specific question on the number of fishers that had drowned within each landing site in the year 2020. The Frame Survey results indicate there were 268 incidents of fisher drowning across 83 landing sites in Kenya. While the frame survey had statistics on drowning incidents, it did not provide information on the socio-demographic profile and occupational dynamics of the victims. This study intended to examine drowning incidents to better understand opportunities for designing appropriate mitigation strategies.

We surveyed 43 out of the 83 landing sites in the Frame Survey (Fig 1) from September 4^th^, 2021, to November 3^rd^, 2021. Beach Management Units (BMUs), the smallest fisheries’ administrative units, are responsible for registering fishers and keeping written records in the landing sites. Within each county and sub-county, we selected the Beach Management Units (BMUs) reporting the largest number of drowning deaths on the Frame Survey. We sought to capture incidents across a range of BMUs in different sub-counties. We detected duplicate fatal drowning incidents in the Frame Survey figures, where BMUs that were geographically close to each other sometimes both counted the same fisher death. We also detected figures that include incidents of fishers who perished over a longer time period outside the study time frame on the Frame Survey (12 months prior to the survey). We targeted incidents linked only to BMUs where reliable reports about the deceased fishers and the circumstances surrounding their deaths were available.

Two data tools were designed to collect data: a semi-structured verbal autopsy questionnaire and a key informant interview guide. At each BMU, we requested the written record of any fisher drowning deaths that occurred over a period of twelve months (July 2020 –June 2021; the year preceding the Frame Survey). For each identified death, the verbal autopsy questionnaire was administered to the BMU member who was familiar with the circumstances of the incident. A verbal autopsy is a tool to collect information about probable causes of death in populations lacking adequate civil registration system with medical certification of cause of death [39–41]. Applications of verbal autopsy are not limited to gathering of data on population cause-of-death structure, but also include exploration of disease outbreaks and risk factors, and investigation of the effectiveness of public health interventions [42–44]. To capture information about fisher drowning death risk factors, our verbal autopsy consisted of four main sections: questions about the person reviewing the incident, characteristics of the deceased (e.g., age, education level, fishing experience), specifics of the incident, and other key risk factors, such as weather conditions (rain, wind), boat maintenance and safety equipment (navigation, life jacket), and use of alcohol or drug. Where the BMU member was unable to provide sufficient information, a close relative or colleague of the deceased, who knew the deceased or who was close to the scene of the incident, was identified to provide information about the deceased and circumstances surrounding the incident. The questionnaires were digitized on Kobo Toolbox.

The leader of the BMU was interviewed using the key informant interview guide. Interviews discussed existing socio-cultural perceptions on fisher drowning and prevailing drowning risk factors. The key informants also provided administrative information and managerial views on the risk management environment, policy framework, and recommendations on possible interventions for hazard mitigation. This research was approved by Cornell University’s Institutional Review Board and participants granted written informed consent.

### Data analysis

To examine the contribution of extreme weather to fisher drowning deaths relative to other risk factors, we analyzed the frequencies with which weather or weather-related variables (heavy rain, strong wind, rough water) and other risk factors (non-use navigation equipment or life jacket, non-motorized boat, inability to swim, use of alcohol or drug, poor boat maintenance) were involved in fisher drowning death incidents. To investigate the relative degree to which other risk factors compound or are compounded by the effect of bad weather, we looked at the co-occurrence of bad weather and other risk factors in drowning incidents. To do that, we calculated the frequencies of other risk factors among the incidents that involved bad weather. Statistical analyses were done with R [45].

Key informant interview responses were thematically coded. A stakeholder analysis was then carried out based on information shared within interviews. A stakeholder analysis is used for designing a program or intervention. The objectives of a stakeholder analysis are to identify the stakeholders of an intervention, their level of influence, their interests, the contribution they can make, and how to engage them [46]. Stakeholders can be individuals, groups, or organizations which can be impacted by the planned intervention or influence its outcomes. The methodology was adapted from the Stakeholder Analysis Matrix Toolkit [46] and Plummer et al. [47].

## Results

### Characteristics of the deceased fishers and their boats

We identified a total of 141 fisher drowning deaths (Table 1). Nearly all the drowning victims in our study were males (140, 99.3%) and most had primary education (70.9%). The average age of deceased fishers was 32.7 years and average household size was 5 people.

**Table 1:** Sociodemographic characteristics of the deceased fishers in Lake Victoria, Kenya.

Variable	Deceased fisher (N = 141)	Percentage or standard deviation
Gender Male Female	140 1	99.3% 0.7%
Age (years)	32.7	9.3
Education None Primary Secondary Other Not available	1 100 23 3 14	0.7% 70.9% 16.3% 2.1% 9.9%
Household size	5.0	3.1

### Drowning risk factors

**Fig 2:**
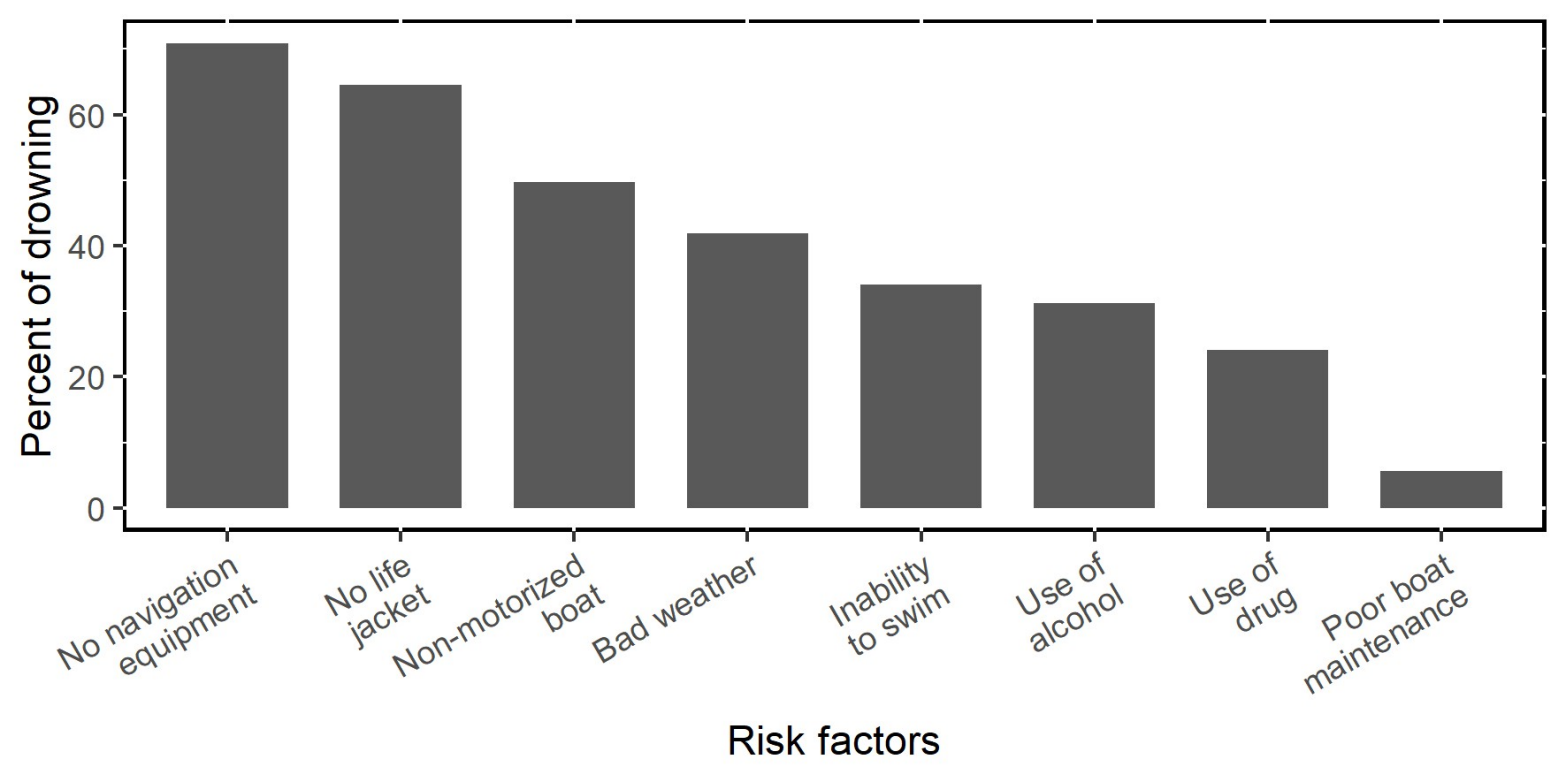
Frequencies of risk factors linked to drowning deaths around Lake Victoria, Kenya.

Bad weather was described as the cause of 41.8% of incidents. Conditions of rough water (47.5%), strong wind (46.8%), and heavy rain (12.1%) were frequently identified as affecting the boats during the incidents (Fig 2, Table 2).

**Table 2:** Reported risk factors linked to drowning deaths around Lake Victoria, Kenya.

Variable	Deceased fisher (N = 141)	Percentage (%)
The incident was because of bad weather Yes No Not available	59 78 4	41.8 55.3 2.8
Water conditions Rough Moderate Slight/Calm Not available	67 31 35 8	47.5 22.0 24.8 5.7
Rain conditions Heavy rain Light rain No rain Not available	17 5 112 7	12.1 3.1 79.4 5.0
Wind conditions Strong wind Moderate wind Little/no wind Not available	66 31 36 8	46.8 22.0 25.5 5.7
Timing of incident		
Nighttime (6pm to 6am)	71	~50%
Daytime (6am to 6pm)	70	~50%
Boat locomotion method Motorized Non-motorized Not available	61 70 10	43.3 49.6 7.1
Boat adequately maintained Yes No Not available	120 8 13	85.1 5.7 9.2
Life jacket or life buoys used Yes No Not available	28 91 22	19.8 64.5 15.6
Communication or navigation equipment used Yes No Not available	18 100 23	12.8 70.9 16.3
Time worked in fishing industry < 3 years 3–10 years > 10 Not available	24 70 36 11	17.0 49.6 25.5 7.8
Ability to swim Yes No Not available	80 48 13	56.7 34.0 9.2
Alcohol used Yes No Not available	44 67 30	31.2 47.5 21.3
Drug used Yes No Not available	34 71 36	24.1 50.4 25.5

Respondents generally reported that boats were adequately maintained (85.1%) when drowning incidents occurred. Half of boats (49.6%) involved in incidents were non-motorized, meaning using sails or paddles. Life jacket or life buoys and navigation or communication equipment were used in only 19.8% and 12.8% of the incidents, respectively **(**Fig 2, Table 2).

Most fishers had substantial experience in the fishing industry at the time of their deaths with more than 75% having fished for over 3 years (Table 2). Only 56.7% were known to be able to swim though. Alcohol (31.2%) and drug (24.1%) use were also common risk factors involved in these incidents (Fig 2, Table 2).

### Co-occurrence of bad weather and other risk factors

**Fig 3:**
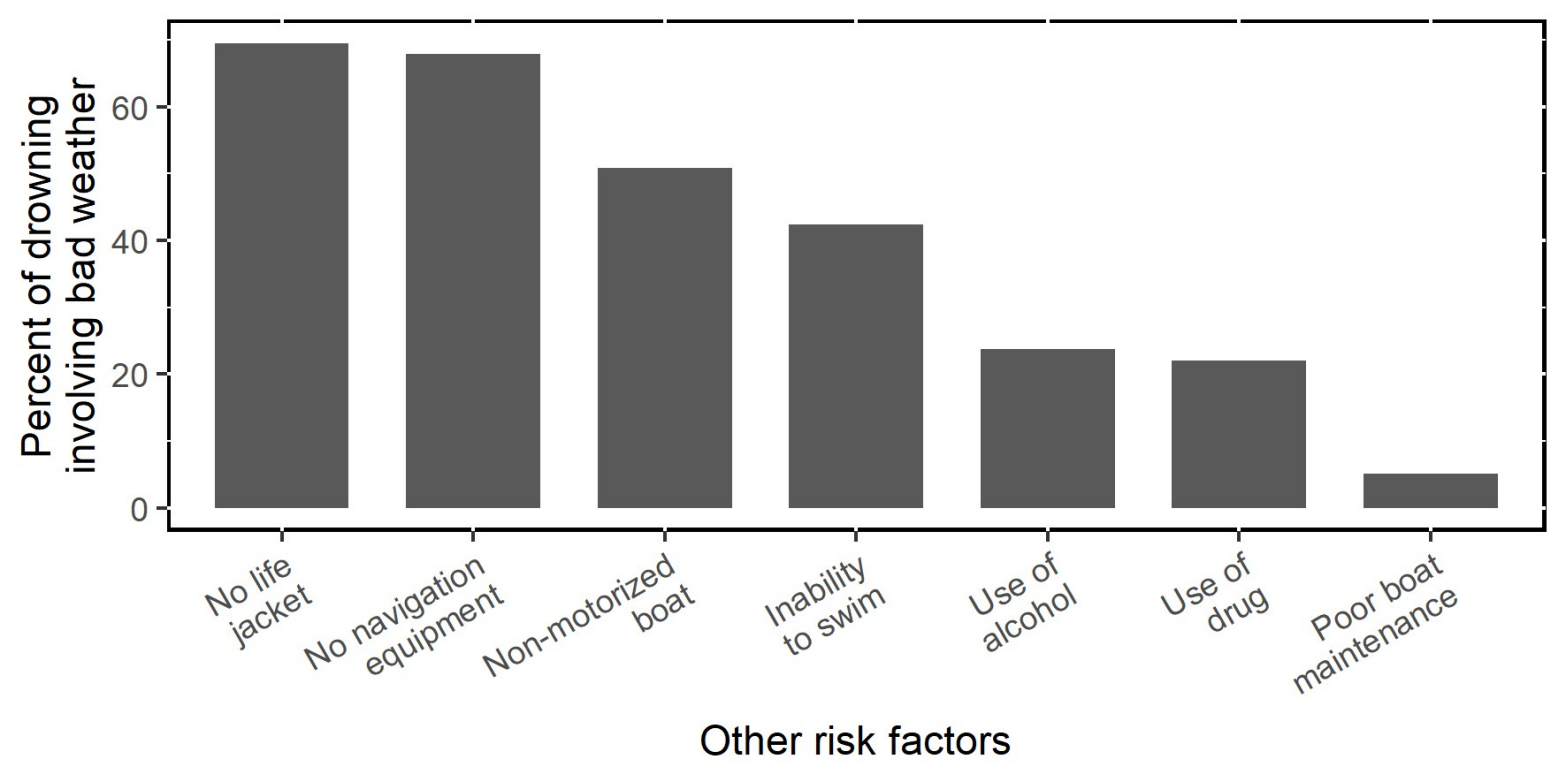
Co-occurrence (%) of other risk factors with bad weather.

Boat and individual risk factors co-occurred with bad weather, meaning multi-faceted risk factors were present. The lowest co-occurrence was with poor boat maintenance (5.08% of fatal incidents involving bad weather also involved poor boat maintenance). The highest was with non-use of life jacket (69.49% of incidents involving bad weather did not use life jacket) (Fig 3).

### Stakeholder analysis

The results of the stakeholder analysis, based on data collected through key informant interviews with BMU leaders, are shown in Table 3.

**Table 3:** Stakeholder analysis matrix around the development of a comprehensive policy on fisher drowning.

Stakeholders	Impact How much does fisher drowning impact them?	Influence/ Power How much influence do they have over fisher drowning?	What is important to the stakeholder?	How could the stakeholder contribute to an intervention?	How could the stakeholder block the project?
**1. Boat Crew**	High	Medium	• Earning a decent income and providing for their families • Safety education and training to reduce risk • Safe vessels and/or responsible boat owners • Affordable, comfortable safety equipment, including life jackets • Accurate, accessible weather forecasting • Protection from crime (e.g., robbery, piracy) • Fast, free search-and-rescue response	• Set example and motivate peers by practicing safe behaviors (e.g., wear life jacket; not use alcohol in lake) • Report unsafe boat condition or insufficient life-saving equipment to relevant authorities, e.g., BMU • Organize (e.g., unions) to negotiate safe conditions with boat owners, and better treatment and support from authorities, etc. • Representatives participate in national water safety plan development	• Disregard safety rules, not use safety equipment, etc. E.g., due to poverty, low risk perception, social norms, conflicting beliefs, discomfort • Overcoming fear of dangers in lake work with additional risks, e.g., by using alcohol/drugs
**2. Boat Owners**	High	High	• Earning income from fishing equipment and/or boat rentals • Affordable and effective materials for boat construction • Meeting legal requirements for safety of boat and its equipment	• Participate in safety trainings • Provide sufficient safety equipment in boats (e.g., life jackets, fire extinguisher, life rings) •Make/purchase/maintain safe boats	• Not provide adequately safe boats, safety equipment, or boat maintenance, e.g., to save money • Fail to properly maintain or repair boats
**3. Boat Makers**	Medium	Medium	• Earning a decent income • Good building materials, e.g., timber • Timely payment by boat owners • Making a boat to meet local standards (craftsmen)	• Participate in training on building safe boats • Build safe boats	• Not build safe boats, e.g., due to insufficient knowledge or motivation
**4. Beach Management Units (BMUs)**	High	High	• Fisher and boat registration and licensing payments • Authority to make beach management decisions • Successful local commerce • Safe practices within the community	• Participate in safety trainings • Raise awareness and advocate for fisher drowning prevention measures with all local stakeholders • Monitor safety regulations and intervene (e.g., with fishers or authorities) when they are not followed • Identify and ameliorate barriers to safe boating and following regulations	• Not monitor, support, or follow-through to ensure boat owners and fishers use safe vessels, equipment, and practices, e.g., due to insufficient knowledge or attention
5. Government a. National and/or County Government (1) Fisheries/water agencies: a) State Dept for Blue Economy b) Kenya Fisheries Service c) Kenya Marine and Fisheries Research Inst. d) Kenya Maritime Authority (2) Kenya Metrological Department (3) Kenya Coast Guard Services (4) Kenya Wildlife Service (5) Kenya Police	Medium	Medium	• Fisheries management, including BMU monitoring and law enforcement at the local level • Protecting and promoting strong, sustainable fisheries • Frequent lake patrols • Enforcement of safety regulations of fishing vessels, and safe behaviors by operators/passengers • Maritime communications (e.g., communication network, rescue coordination and training, and weather alerts) • Timely, accurate weather forecasts from Kenya Meteorological Department • Education and awareness of (a) fisheries and transport laws, regulations; (b) use of weather information; (c) small vessel operation, including water safety education, boat standards, emergency response	• Provision of basic water safety education • Support for access to weather information • Regulation enforcement, including patrols and inspections, including high-risk, remote areas, to better support fishers and protect them from unsafe vessels and practices • Drowning prevention intervention evaluation (e.g., involving KMFRI) • Fast, free search and rescue responses • At the ministry level, participate in development and implementation of water safety plan	• Not monitor, support, or follow-through to ensure fishers use safe vessels, equipment, and practices, e.g., due to insufficient capacity/resources, disinterest, corruption, or conflict of interest
b. Regional Government (e.g., Lake Victoria Fisheries Organization, Lake Victoria Basin Commission)	Medium	Medium	• Establish communications system for safety on Lake Victoria and an East African Maritime Transport Strategy • Collect data to monitor changes in drowning risk factors and mortalities	• Collaborate in advocacy and agenda-setting to reduce fisher drowning in Lake Victoria	• Not engage in or prioritize fisher water safety initiatives

Adapted from: Tools4Dev [46] and Plummer et al.[47]

Our stakeholder analysis provides insights into the details underlying the risk factors in fisher drowning death incidents, and potential solutions to mitigate the risks. Fisher drowning death related to bad weather, for example, can be due to ineffective or inaccessible weather warning systems, poverty (insufficient income), low risk perception, or a combination of these factors. Possible mitigation strategies include the development of timely and accurate weather forecasts by the Kenya Meteorological Department and governmental support that ensures boat crews access weather information in a timely manner.

Fisher drowning deaths related to boat and equipment-related risk factors (non-use of navigation equipment and life jacket, poor boat maintenance) can stem from disregard of safety rules by boat crews, which in turn can be due to poverty, low risk perception, social norms, and beliefs. Risk factors related to boat and safety equipment can also originate from power structures of boat ownership and labor, with boat owners not providing safe boats and safety equipment to save money or simply because of lack of knowledge or motivation. Examples of identified mitigation strategies are training (including by peers), support to boat operators (crews, owners, makers), awareness campaigns, regulation monitoring, evaluation, and enforcement.

For BMU and government stakeholders, high level items that affect drowning risk factors include management (e.g., boat registration and licensing), awareness campaigns, monitoring, evaluation, regulation, enforcement, support to local stakeholders (i.e., boat crew, owners, and makers), and a system to ensure communication between the governments of the three countries bordering Lake Victoria (Kenya, Tanzania, Uganda). It is worth noting that the needs for training were identified for different levels of stakeholders.

## Discussion

Bad weather was implicated as a cause of drowning deaths in 41.8% of the incidents, with strong winds (46.8%) and rough water conditions (47.5%) often implicated. Fishers may have been caught in unexpected or quickly changing weather conditions or may have gone fishing despite poor weather. Indeed, many fishers have limited savings and face a complex constellation of risk calculations, including that income is directly tied to fishing effort. Prolonged periods of bad weather may thus particularly exacerbate risks as the need to generate income pushes people to fish in risky conditions. While our study did not estimate the fisher drowning incidence, previous studies in Lake Victoria have suggested fishers face a 1.4% risk of drowning deaths [3] and indicated events of nearly capsizing (57.8%) and actually capsizing (21.7%) are shockingly common [10]. Our findings indicate that actions across multiple levels of stakeholders are imperative to improve water safety skills and utilize multiple safety measures to prevent fisher drowning deaths.

Communities in the Lake Victoria basin lack effective weather advisory and warning systems [21]. However, our stakeholder analysis and other studies in the Lake Victoria region [21, 48] indicate that weather warning systems present an opportunity to counter drowning risks in response to escalating climate change-related extreme weather events [9, 13, 14]. Examples from East Africa indicate that the design of these systems requires close collaboration between weather service offices, BMU officials, and community stakeholders [21]. As noted within our stakeholder analysis, fishers are balancing a complex set of risks and dependent on fishing for income. Water safety risks are coupled with risks related to insufficient income if fishing is skipped. Weather warnings would thus need to be timely and well-tuned in accuracy to convince fishers to forgo income. Combining state-of-the-art technology (by experts in weather service offices) with field observations (provided by community stakeholders) can ensure forecasting accuracy [21]. Use of various means of communication (e.g., radio broadcasters, local intermediaries, and via smartphones) can also ensure forecasts reach targeted users within fishing communities [21]. Alongside warning systems, there would also ideally be a safety net for fishers to access alternative livelihood opportunities during periods of bad weather.

Our study revealed few fishers had life jackets (19.9%) or communication or navigation equipment (12.8%), and just over half knew how to swim (56.7%). Previous studies have found similar rates of use of life jackets and communication tools, and swimming ability in fishing communities around Lake Victoria [3, 10]. These risk factors (non-use of life jacket and navigation equipment, inability to swim) also co-occurred with bad weather at high rates (69.5%, 67.8%, and 42.4%, respectively) to jointly contribute to fatal drowning incidents. Such co-occurrence of risk factors suggests that fisher drowning deaths are often the result of constellation of risk factors coming together and cross-sectoral actions are imperative to mitigate the effect of extreme weather events, which are predicted to increase in intensity and frequency as the world’s climate becomes warmer [8].

In line with our findings, a study in Lake Albert, Uganda (one of the three countries bordering Lake Victoria), indicates that perceived high costs of life jackets, limited knowledge, social norms and beliefs (e.g., distrust in jackets’ effectiveness, belief that it is women who should wear life jackets) were among the barriers of using life jackets [49]. To address costs, we suggest that targeting boat owners, as opposed to individual fishers, as those responsible to ensure life jacket availability may be the most salient approach given their higher incomes and norms around vessels needing to provide safety equipment. Studies in Lake Albert also suggest that enforcement, peer-led awareness campaigns and training were effective strategies to increase the use of lifejackets [49–52]. Encouraging fishers to motivate their peers’ compliance with regulations on life jacket use, as indicated in our stakeholder analysis, therefore represents a key opportunity to improve safety.

While boats were largely seen as well maintained, motorized boats involved in drowning incidents (43.3%) was surprisingly high relative to the motorization rate of the entire Kenyan Lake Victoria fleet; just 16.5% of the fleet was motorized in the 2014 Lake Victoria Frame Survey [15], though this number has likely risen. This finding suggests motorization may increase risks, with motorized vessels potential to travel further exposing them to greater risks in weather changing over time and distance from shore slowing rescues. Our findings suggest focusing on compliance (regulation and enforcement) among motorized vessels may be particularly valuable.

Fisher drowning victims were of an economically productive age (average of 33 years old), highlighting the potential for far-reaching societal implications of the loss of these individuals. Findings from the present study corroborates those of Kobusingye et al. [10] and Whitworth et al. [3] who found that the majority of drowning victims in the Ugandan and Tanzanian sides of Lake Victoria were under the age of 40. Males made up the bulk of our sample, which was expected given that fishing is dominated by men [15]. This finding is also consistent with several studies that suggest that the burden of occupational drowning is disproportionately high among men [53].

Our stakeholder analysis underlines how careful engagement across groups will be imperative to designing effective risk mitigation tools. This is critical since decisions about whether to fish are made at multiple levels. BMUs permit fishing at an institutional level, boat owners act as managers of fishing crews and employ laborers, and individuals participate in fishing crews. Across these multiple levels, water safety and responses to storm warnings need to be aligned, normalized, and prioritized to improve fisher safety. Discussion across these stakeholders of findings regarding fisher drowning deaths and opportunities to improve safety will be a critical next step. The strategies that are taken up could respond to identified risk factors in a range of intensities, often described as ‘levels of intervention’ [54]. Levels of intervention range from monitoring the situation (e.g., collecting more detailed data on drowning deaths, particularly as weather conditions shift) and communication strategies (e.g., making fishers aware of key risk factors) to eliminating or restricting choices (e.g., sharply restricting fishing in certain weather conditions or mandating vessel safety gear).

Many of the recommendations of the World Health Organization on drowning prevention (WHO) and the United Nations (UN) resolution on global drowning prevention [55, 56] are supported by our stakeholder analysis, which also emphasizes the need to tailor the implementation of these global recommendations to local circumstances. Education and training are identified across stakeholders. Because, globally, the highest drowning rates are among children, a WHO recommendation focuses on teaching school-age children swimming, water safety, and safe rescue skills [55]. Introduction of swimming, water safety, and first-aid lessons as part of school curricula, as recommended by the UN resolution on global drowning prevention [56] that particularly targeted fishing communities around Lake Victoria could reduce child drowning deaths in the short term and fisher drowning deaths in the long term as trained children become fishers; given that 89% of the deceased fishers had at least primary school education this may be a particularly promising strategy. Extension of such training programs to current fishers in small-scale fishery dependent communities around Lake Victoria, or to similar ones in low- and middle-income countries where fisher drowning death rates can exceed those of children [3] and large proportions of drowned fishers cannot swim (e.g., 34% of the incidents we reported), will also be a valuable opportunity to address drowning deaths.

Strengthening of public awareness through strategic communications and enforcement of boating regulations [55] was also identified across the stakeholders. Strategic communications can include a combination of traditional and modern means (radio broadcasters, local intermediaries, and via smartphones, peers) as relevant in different communities [21]. Enforcement must involve engagement across stakeholders to be effective (e.g., fishers report unsafe boat condition or insufficient life-saving equipment to BMUs; BMUs monitor safety regulations and intervene when they are not followed; authorities enforce regulation, including patrolling and inspection). Development of a national water safety plan [55, 56] was also identified for boat crew and government stakeholders.

Regional government (e.g., Lake Victoria Fisheries Organization, Lake Victoria Basin Commission) cooperation in tackling fisher drowning deaths, as recommended in the UN resolution, could facilitate sharing of lessons learned and best practices [56]. Coordination of data collection across governments could also improve policy evaluation.

Compared to other studies on drowning deaths in Lake Victoria fishing communities [3, 10], we specifically focused on small-scale fishers and weather-related risk factors. Our study is therefore more sensitive to specific information about a climate-change vulnerable occupational group. Another key strength of our study is the careful comparison of descriptions of each drowning victim and incident, enabling the removal of duplicated reports or reports that extended outside the timeframe of our study. We, however, may have missed some risk factors. Boat stability, for example, can be a factor that determines the outcome of fishers’ encounters with storms that we did not collect information on. Note, however, that boats used by small-scale fishers in Lake Victoria are usually small [21] and thus unlikely to be stable in heavy storms. Furthermore, some of the behavioral information we collected can be sensitive (e.g., use of alcohol, drug, and non-use life jacket). Respondents may have not been willing to admit to the involvement of deceased loved ones in such a behavior, even if guaranteed anonymity. This sensitivity issue may explain the high percentage of missing data for use of alcohol, drug, and life jacket (21.3%, 25.5%, 15.6%, respectively), though these may also have been reasonably unknown by the respondent, especially if they were not fishing together. The discrepancy of observed and self-reported life jacket wear (0.7% and 31.9%, respectively) in fishing communities in Lake Albert, Uganda [50], reveals the potential sensitivity of the issue in fishing communities in East Africa.

## Conclusions

As climate change alters our environment, it will also reshape the risks faced by the people most reliant on it. Occupational drowning among fishers represents a little examined issue that may have a substantial impact on small-scale fishing communities reliant on coastal and inland waters. Escalating climate extremes, however, come alongside longstanding inequities in access to safety equipment (e.g., life jackets, navigation equipment) and intensifying fishing pressure to maintain harvests. This formidable combination of risks necessitates both careful monitoring of the situation and proactive development of strategies to mitigate occupational drowning risks for small-scale fishers.

## Supporting information

S1 Checklist (DOCX)
